# IUC26678-94 Impact of Metastatic Patterns on First-Line Therapy in mRCC: Meet-URO 33

**DOI:** 10.1093/oncolo/oyag286.023

**Published:** 2026-08-21

**Authors:** E Peretto, S E Rebuzzi, E Verzoni, S Buti, M Di Napoli, C Lolli, M Fanelli, C Masini, P A Zucali, A Mennitto, M Sorarù, R Filippi, L Formisano, C Messina, F Maines, G Fornarini, S Chiellino, L Bonomi, D Bimbatti

**Affiliations:** University of Turin - Italy; Medical Oncology Unit 2, Ospedale Molinette, Azienda Ospedaliero-Universitaria Città della Salute e della Scienza di Torino, Torino, - Italy; Oncologia Medica Genitourinaria, Fondazione IRCCS Istituto Nazionale dei Tumori, Milan, - Italy; Medical Oncology Unit, University Hospital of Parma, Parma, Italy; Medicine and Surgery Department, University of Parma, Parma, - Italy; Department of Urology and Gynecology, Istituto Nazionale Tumori IRCCS Fondazione G. Pascale, Naples - Italy; Department of Medical Oncology, IRCCS Istituto Romagnolo per lo Studio dei Tumori (IRST) “Dino Amadori”, Meldola - Italy; Department of Medicine (DAME), University of Udine, Udine - Italy; Medical Oncology. Comprehensive Cancer Centre, AUSL-IRCCS di Reggio Emilia, Reggio Emilia - Italy; Department of Biomedical Sciences, Humanitas University, Pieve Emanuele, Milan, Italy; Department of Oncology, IRCCS, Humanitas Clinical and Research Center, Rozzano, Milano - Italy; Department of Medical Oncology, Azienda Ospedaliera Universitaria “Maggiore Della Carità”;, Novara - Italy; U.O. Oncologia, Ospedale di Camposampiero, Camposampiero - Italy; Medical Oncology 1U, Azienda Ospedaliero-Universitaria Città della Salute e della Scienza di Torino - Italy; Department of Clinical Medicine and Surgery at University of Naples Federico II, Naples - Italy; Ospedale Arnas Civico, Clinical Oncology, Palermo - Italy; Medical Oncology Department, Santa Chiara Hospital, Trento - Italy; Medical Oncology Unit 1, IRCCS Ospedale Policlinico San Martino, Genoa - Italy; Medical Oncology Unit, IRCCS Policlinico San Matteo, Pavia - Italy; Medical Oncology Unit, ASST Papa Giovanni XXIII, Bergamo - Italy; Oncology 1 Unit, Istituto Oncologico Veneto IOV - IRCCS, Padua - Italy

## Abstract

**Background:**

Metastatic site is a well-recognized prognostic factor in metastatic renal cell carcinoma (mRCC). Endocrine metastases, particularly to the pancreas and thyroid, are associated with favorable outcomes, whereas bone, liver, and brain metastases are linked to poorer prognosis. However, whether these metastatic patterns influence first-line treatment selection remains unclear.

**Methods:**

Meet-URO 33 is an ongoing Italian multicenter retrospective/prospective observational study including patients with mRCC treated with first-line immune checkpoint inhibitor plus immune checkpoint inhibitor (ICI–ICI), immune checkpoint inhibitor plus tyrosine kinase inhibitor (ICI–TKI), or tyrosine kinase inhibitor (TKI) monotherapy. Patients were classified as having favorable-prognosis metastases (pancreatic and/or thyroid only) or poor-prognosis metastases (bone, liver, and/or brain only). Associations between metastatic patterns, treatment selection, progression-free survival (PFS), and overall survival (OS) were assessed.

**Results:**

Among 2,027 enrolled patients, 189 (9%) had favorable-prognosis metastases (pancreas 8%, thyroid 1%), while 1,022 (50%) had poor-prognosis metastases (bone 30%, liver 15%, brain 5%). Patients with favorable-prognosis metastases more frequently received first-line TKI monotherapy than ICI–TKI or ICI–ICI (17% vs. 10% vs. 3%; *p*<0.001). Conversely, patients with poor-prognosis metastases were more commonly treated with ICI–TKI than ICI–ICI or TKI alone (50% vs. 38% vs. 33%; *p*<0.001). On multivariable analysis, favorable-prognosis metastases were independently associated with improved OS (HR 0.34, 95% CI 0.19–0.61; *p*<0.001) and PFS (HR 0.53, 95% CI 0.37–0.76; *p*<0.001), whereas poor-prognosis metastases were independently associated with worse OS (HR 1.32, 95% CI 1.11–1.57; *p* = 0.002) and PFS (HR 1.20, 95% CI 1.04–1.38; *p* = 0.013).

**Conclusions:**

Favorable- and poor-prognosis metastatic patterns are associated with distinct survival outcomes and different first-line treatment choices in mRCC. Although the observational design precludes causal inference, TKI-based strategies appear to play a central role in both settings. TKI monotherapy is more frequently used in patients with favorable-prognosis metastases, likely reflecting their indolent and angiogenic biology, whereas ICI–TKI combinations are preferred in poor-prognosis disease, where tumor shrinkage and disease control are priorities. Prospective studies are warranted to determine the predictive value of these metastatic patterns across different treatment strategies.

